# Supplementary figures and images for: Real-Life Cefiderocol Use in Bone and Joint Infection: A French National Cohort
Source: Antibiotics (Basel). 2025 Apr 8;14(4):388. doi: 10.3390/antibiotics14040388 (PMC12023946; doi:10.3390/antibiotics14040388)

Figure S1. MIC distribution.

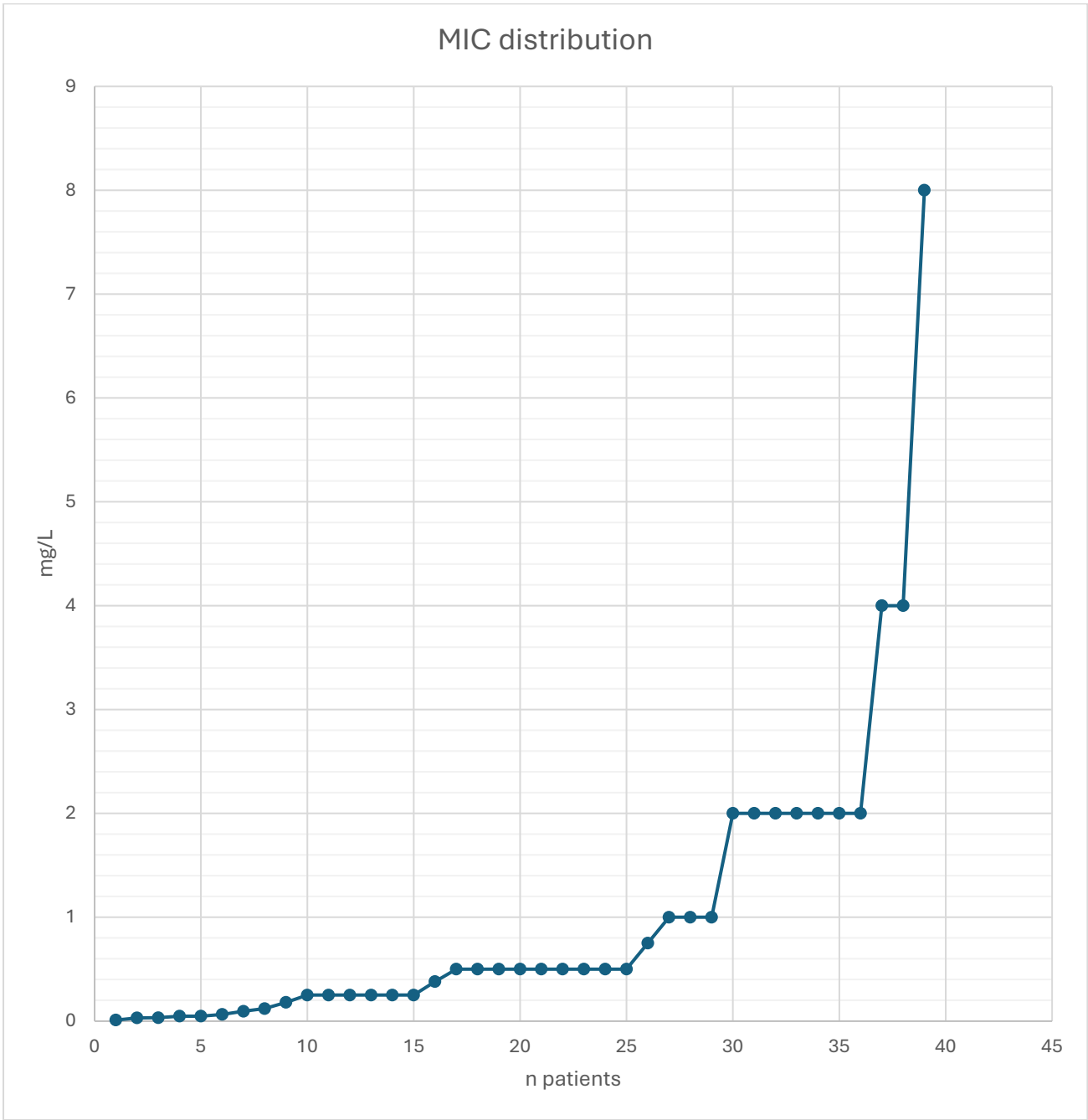

Supplement: Supplementary file 1 [file antibiotics-14-00388-s001.zip › antibiotics-3374477-supplementary.pdf]
